# On Phrenology

**Published:** 1822-06

**Authors:** 


### CONJECTURAL PHYSIOLOGY.*

### Art. VIII.—*On Phrenology.*

THAT there is nothing more conducive to the quick and thorough knowledge of a contested subject, than the candid investigation of its merits by as many persons as may be found competent to the task, will be readily allowed; and, no less readily, that nothing tends more to discourage such investigation, and bring unmerited contempt upon a subject, than to see a paper ridiculing it, from the pen of a member of a liberal profession, evidently a man of talent, inserted in a public and professedly-scientific Journal. Since the first promulgation of the opinions of Drs. Gall and Spurzheim, their system has been the theme, alternately, of commendation and abuse; and but few, comparatively, have been found both willing and able to examine and illumine so intricate a subject. The few who have, it is true, (and all of them rank high as men of great mental endowments,) are, without one exception, convinced of its correctness, or, at least, confess themselves unable to substantiate any objection to it. This, however, has not deterred others, evidently less acquainted with it, from denouncing the system altogether as untrue, and averring, they do not hesitate to affirm "that instances at variance with the doctrines of phrenology, are daily crossing our path;" though they have not, at present, brought one forward for public examination.

The paper to which these strictures more particularly allude is one contained in the London Medical Repository for April, by "Mr. Edward Thomas Bond, Surgeon;" which, though extremely alluring, adorned as it is by poesie and rhetoric, will, divested of these, I do not fear being able to show, appear a train of sophistry and error. Mr. B. starts by telling us, there are few unbiassed by authority. Then, supposing the system possibly true, he congratulates his readers, very facetiously, upon possessing "a thermometer of talent, by which we may readily judge to what height the mercury of genius may have risen." So far no comment is necessary; but he now becomes alarmed by the "more serious demands made upon our credulity;" for phrenologists require him to believe "that the brain is an aggregate of many organs; that a trivial enlargement of it [one of them,] possesses powers not in common with the mass of which it is in continuity, of whose nature it partakes, and whose anatomical structure it resembles and is in common with its own, but powers peculiar to itself, though appropriated

* We adopt this expression in contradistinction to what may be called *positive* physiology, founded on facts and fair induction. *Conjectural* physiology is, to the natural history of man, what metaphysics are to its moral consideration.—A. B. G.

by mere position,—powers which establish its unity, and exalt it at once into an individual and specific existence."

I will now inquire of Mr. B. why he should deny to the brain the same characteristics which are so readily allowed to its active principle, or the active principle resident in it (the mind)? What would be the answer of metaphysicians or theologists, were the following questions put to them:—Is the mind an aggregate of distinct perceptions, faculties, and ideas, or not?—Would the better education of one of these faculties render it more extensive and powerful or not? Answers to these questions would, in our present state of knowledge, be certainly in the affirmative: then why should not each have a peculiar habitation, and be arranged with the same order and regularity as the rest of Nature's works? and why should not that habitation be increased proportionally with the development of the faculty? These must be allowed, if we reason at all from analogy; but the numerous *facts* collected by the professed investigators of this science render all hypothetical reasoning unnecessary.

He goes on:—" That, according as the predominant development is situated here or there, every individual is impressed, at his birth, with the particular tendency which such development is asserted to produce; and, in truth, that we are all, and each of us, at our entrance into life, hurried (or, if that is too strong a term, predisposed,) by our very make, to acts of benevolence or of murder,—of justice or of theft: an extension in this quarter inclining Turpin to depredation, just as an evolution in that stimulates a Howard to philanthropy, (I am careful not to exaggerate;)" and culpably careful of exposing a fundamental proposition of the system, which expressly says, " our actions depend upon the balance of power between the organs most developed;" except by insinuating it placed there by phrenologists, " to coax a compliance with their opinions;" and entirely overlooking the assistance given by education, &c. to the side of morals. Mr. B. now supposes cases, which, being the counterparts of others long since refuted by abler pens than mine, I shall pass over entirely. (See COOMBE, ABERNETHY, and *New Edinburgh Review*.) It is, then, taken for granted the system will exert a demoralizing influence. He says, " Who, amongst the criminals, would not lay it 'as a flattering unction to his soul' that he was born with a predisposition to the very guilt into which he has plunged?"—" It is true," he says, " the favourers of craniological philosophy insist, this system will exert upon society an influence the reverse of prejudicial: but confidence of assertion is not the criterion of truth." Mr. B. then closes with some propositions answered long ago, and two pages of poetical rhapsody, " signifying nothing."

I shall now no longer intrude upon these pages, than by quoting a few lines from Sir G. M'Kenzie.—"It cannot be too often impressed on the student of phrenology, that it is *impossible* to know, by external signs alone, the *character* of any individual. We can only ascertain what dispositions he possesses most strongly; and, by *long observation* of his actions and conversation, we may discover whether he has subdued the lower propensities, and given due exercise to the higher faculties. We may, after a little practice, observe the kind, and also the degree, of talent possessed by an individual; but it is impossible to ascertain, by simple inspection, whether he has, or has not, misapplied his talents; or even whether his feelings and propensities be active or otherwise. By observing proportions, we may, however, judge to what conduct he is naturally prone; but we can never pretend to predict actions."

JUNIUS.*

*April 27th*, 1822.

* It should have been, we apprehend, *Junior*. The paper itself is very *juvenile;* and Junius could never have raved about cranioscopy, however shrewdly he may have judged of the value of certain people's brains.—A. B. G.

